# Coping mechanisms and resilience in psychiatric trainees during COVID-19 pandemic

**DOI:** 10.1192/j.eurpsy.2021.697

**Published:** 2021-08-13

**Authors:** C.-A. Crisan, R. Pop, A. Mihai

**Affiliations:** 1 Psychiatric Clinic 1, Emergency County Hospital Cluj-Napoca, Cluj-Napoca, Romania; 2 Neurosciences, Iuliu Hatieganu University of Medicine and Pharmacy Cluj-Napoca, Cluj-Napoca, Romania

**Keywords:** coping mechanisms, resilience, Covid19 pandemic, stress

## Abstract

**Introduction:**

The Covid-19 pandemic has a profound impact on all domains of day to day life, forcing individuals to make substancial change in the way of living. Such change is known to cause an important psychological distress, and in some persons evidencing silent disorders among apparently functional individuals. Good coping mechanisms and resilience can be the key to overpass this difficult period.

**Objectives:**

The aim of this study is to evaluate the coping mechanisms and resilience that Romanian psychiatric trainees used during Covid19 pandemic.

**Methods:**

We developed an online questionnaire. We included questions about different socio-demographical variables and about coping mechanisms (using COPE scale), resilience (using Connor-Davidson Resilience Scale) and quality of life (using QoL Scale).

**Results:**

The preliminary data show that staying busy, seeking social support and having a positive minset are emotion-focused coping strategies present in individuals who overpass easier this period.

**Conclusions:**

The Covid-19 pandemic is creating significant distress and impairment in functioning, but individuals who have good psychological mechanisms and who are more adaptable are less vulnerable during Covid-19 pandemic. Future research should build upon these findings to better understand coping mechanisms during crises and also social policies should be developed to acknowledge the variable needs in adults.

